# Understanding the Basis for Down Syndrome Phenotypes

**DOI:** 10.1371/journal.pgen.0020050

**Published:** 2006-03-31

**Authors:** Randall J Roper, Roger H Reeves

## Abstract

Down syndrome is a collection of features that are caused by trisomy for human Chromosome 21. While elevated transcript levels of the more than 350 genes on the chromosome are primarily responsible, it is likely that multiple genetic mechanisms underlie the numerous ways in which development and function diverge in individuals with trisomy 21 compared to euploid individuals. We consider genotype–phenotype interactions with the goal of producing working concepts that will be useful for approaches to ameliorate the effects of trisomy.

## Introduction

Trisomy 21 occurs in 1/750 live births. The frequency of Down syndrome (DS) is much higher at conception, given that up to 75% and 50% of DS fetuses identified during the first and second trimester, respectively, are lost before term [1,2]. Trisomy for some other autosomes occurs more frequently than trisomy 21, nearly always resulting in prenatal loss [3]. The relatively high frequency of postnatal survival for trisomy 21 is thought to be principally a function of the small number of genes on human Chromosome 21 (Hsa21), the smallest and least gene-dense of the autosomes.

## Phenotypes

The clinical presentation of DS is complex and variable. A few features occur to some degree in every individual with trisomy 21, including characteristic facial dysmorphology, a small and hypocellular brain, and the histopathology of Alzheimer disease, which is present by the fourth decade. Individuals with DS are invariably cognitively impaired, though the severity is highly variable. Hypotonia occurs frequently in newborns, and most have atypical dermatoglyphic features, though the specific subset of these is again individually variable.

Trisomy 21 is also a risk factor for a number of diseases. For example, it is among the leading causes of congenital heart disease (CHD), some form of which occurs in 40%–50% of those with DS [4]. The incidence of childhood onset leukemia and Hirschsprung disease are both significantly elevated in individuals with trisomy 21. Health-care guidelines for individuals with DS include more than 80 clinical features that occur more frequently than in the population at large [5]. Three critical points for this discussion arise from these basic observations: (1) the incidence of most phenotypes seen in DS is variable; (2) the severity of a given feature is highly variable; and (3) none of the features diagnosed in DS is unique to people with trisomy 21. For “DS features” that also occur in euploid individuals, we assume that there is some commonality of etiology regardless of ploidy, but this must be proven for any specific case.

A central challenge of genetic research in humans is to precisely define phenotype. This is especially critical in DS, which is a product of genetic effects on different cells, structures, and functions throughout development, many of which may have cascading effects to produce clinically observed phenotypic end points in a given individual with trisomy 21 [6]. A first step in this process is to separate those effects of trisomy that disturb development from those that alter function of cells that have reached an end point of differentiation. These are obviously not independent concepts; any “developmental” perturbation derives from alteration of some function in a developing cell. However, understanding when trisomy causes a divergence from normal patterns of development in a cell that exists only for a defined period during embryogenesis requires a different experimental approach (and, ultimately, a different therapeutic approach) than measuring how trisomy affects a steady-state function (e.g., a signaling or metabolic pathway, neuronal response to stimulation, etc.) in a terminally differentiated cell. Indeed, the altered functions of a mature cell may have little or nothing to do with up-regulation of trisomic genes in that cell, but rather could reflect a developmental error caused by trisomy that has downstream consequences that affect function. That is, a specific phenotype may be a *consequence* of but not a *direct product* of trisomic gene expression (developmental versus functional effects).

## Genetic Models for DS

Because understanding the impact of elevated gene expression throughout development is essential in DS research, animal models play a critical role, especially for correlating the direct and cascading effects of trisomic gene expression on development and function. The best-characterized mouse models to date are trisomic for segments of mouse Chromosome 16 (Mmu16) conserved with Hsa21. The Ts65Dn mouse is trisomic for a segment that contains orthologs of about half of Hsa21 genes while Ts1Cje mice are trisomic for about two-thirds of the genes that are trisomic in Ts65Dn [7,8]. A variety of DS phenotypes have been assessed quantitatively in these models, providing the basis for tracing their origins in development.

Trisomic gene content can be manipulated by chromosome engineering to add or subtract trisomic segments in mice [9]. Recently, a transchromosomal DS mouse model was reported that inherits a copy of a nearly intact Hsa21 [10]. While mosaicism due to random loss of the human chromosome from subsets of mouse cells during development represents an important consideration in making genotype–phenotype correlations in these mice, the gene content of the cells that remain trisomic provides a nearly ideal representation of the genetic condition in DS. Indeed, these mice demonstrate a number of developmental problems analogous to those in DS, including similar defects in heart development that are not seen in the models with trisomy only for Mmu16 orthologs of Hsa21 genes.

Manipulating the set of genes that are trisomic in a mouse can be used to build powerful models. The availability of complete genome sequences for Hsa21 and its mouse orthologs supports a gene catalog to further understand the genetic contributions to DS phenotypes in the mouse. These models provide one of the few ways to systematically study the prenatal consequences of trisomy 21.

## Mechanisms of Gene Action

**Figure 1:**
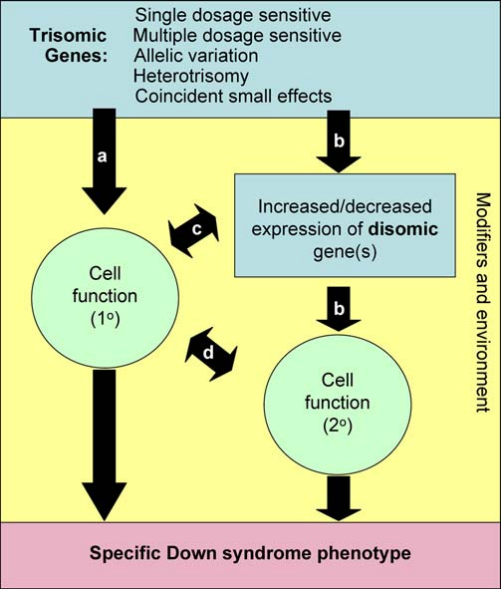
Possible Phenotypic Consequences of Gene Action in Down Syndrome (A) A trisomic gene or genes might directly affect cellular function in a fully differentiated cell to cause a functional phenotype of DS or in an immature cell to produce a developmental phenotype. (B) Trisomic genes may alter expression of disomic genes, leading to a cellular manifestation and a DS phenotype. A trisomy-induced change in cellular function altering the relationship of that cell to surrounding cells leads to a secondary distortion of (C) disomic gene expression or (D) function in neighboring cells. Modifier genes or environment (yellow box) might interact at multiple points to initiate, ameliorate, or exacerbate phenotypes.

Mouse models of DS show elevated expression of most triplicated genes across a wide range of tissues throughout development, maturation, and aging [11,12]. The ways in which genes that are present in three copies might contribute to changes in cell function directly or by modification of disomic gene expression to cause specific DS phenotypes is likely to represent the full range of genetic mechanisms seen in other complex traits, with some additional aspects specific to trisomy (Figure 1). We consider here effects of single dosage-sensitive genes, alone or in combination; the possible contributions of multiple recessive alleles and heterotrisomy; small additive or coincident effects of dozens of genes; and roles for disomic modifier genes.

### 

#### Dosage-sensitive genes.

The simplest model for gene action in DS is of a single dosage-sensitive gene that acts by itself to produce a phenotype, independent of effects by other genes or the environment to either buffer or exacerbate its dosage effect. In this sense, the gene is Mendelian in its function. A number of transgenic mice have been engineered to express elevated levels of Hsa21 genes or their mouse orthologs (see [13]). For the most part, these models have not been used to compare quantitatively the phenotypes in mice with segmental trisomy for the same (plus other) genes. A number of early transgenic studies used constitutive promoters to obtain high levels of expression without regard to normal spatial and temporal patterns for that gene. These types of studies may provide some insights into possible roles for a gene, but they are at best several steps removed from the conditions that produce specific phenotypes in DS.

The single dosage-sensitive gene model underlies hypotheses of “critical regions” on Hsa21, chromosome segments believed to include a dosage-sensitive gene or genes that are responsible for a given aspect of the DS phenotype. Shortly after the discovery of trisomy 21 as the cause of DS in 1959 [14], rare individuals with partial trisomy 21 were identified who had two complete copies of Hsa21 and a third copy of a subset of genes from this chromosome due to cytogenetic rearrangements [15,16]. Comparison of the triplicated regions from individuals who shared a given phenotype of DS could sometimes identify a common region of overlap believed to contain the “critical” gene(s) in a Down syndrome critical (or chromosomal) region (DSCR). The best-described DSCR extended about 5.4 Mb on Hsa21 [17,18]. This region was associated with several of the major DS phenotypes, including protruding tongue and flat facies (largely a function of hypoplastic mandible and craniofacial skeleton, respectively), short stature, mental retardation, joint hyperlaxity, muscle hypotonia, and a variety of dermatoglyphic abnormalities. The DSCR hypothesis predicted that a gene or genes in this region were sufficient to produce these DS features when present in three copies.

Several features attributed to this DSCR have direct parallels that can be measured precisely in Ts65Dn mice. With these phenotypic “readouts” for the predicted functions of a critical region gene or genes, Olson et al. [9] made a critical region model by re-engineering Mmu16 such that the region corresponding to this DSCR was duplicated or deleted. Mice carrying the duplication, which had segmental trisomy involving only the critical region genes, did not display the effects on stature nor the midface hypoplasia, small mandible, or dysmorphology of the skull predicted by the DSCR hypothesis. Thus, no gene(s) from this region was sufficient to produce these phenotypes. Next, mice deleted for the critical region segment were crossed to Ts65Dn mice (which display all of these DS characteristics), thus returning critical region gene dosage to normal in an animal that carried the majority of Ts65Dn segment genes in three copies. These mice had a somewhat attenuated presentation of phenotypes seen in Ts65Dn, indicating that while critical region genes made some contribution when present in three copies, they were largely not necessary for these effects. This result suggests that for those specific phenotypes, the DSCR hypothesis of single gene effects is not correct. Rather, multiple genes are required to produce these complex alterations to structures that are the products of intricate developmental processes.

Some aspects of DS may in fact be due primarily to the effects of a single dosage-sensitive gene on Hsa21. For example, elevated expression of endostatin, a protein that inhibits angiogenesis required for tumor growth, may explain at least part of the cancer resistance seen in DS [19]. However, it seems to us unlikely that many aspects of the DS phenotype that show highly variable presentation and derive from changes in structures that are the product of a long span of development are likely to reflect the effects of a single dosage-sensitive gene. Indeed, the classical understanding of Mendelian “single-gene” mutations as independently acting elements has been qualified with the greater appreciation for the roles of modifier genes on the phenotype.

#### Interacting genes of major effect.

A simple extension of the single dosage-sensitive gene model is to imagine additive effects of multiple dosage-sensitive genes interacting in a specific cell type during development. This could occur due to co-expression of two or more genes of major effect in the same cell at the same time or at different stages in the developmental history of that cell population. The effects of multiple dosage-sensitive genes might be amplified (or attenuated) when they occur within the same biochemical pathway. Possible trisomy 21 effects on a number of pathways have been posited [20], prioritizing them as targets for molecular analysis. However, the functions and interactions of most Hsa21 (and other) genes are not catalogued to this level. The combinatorial possibilities for testing groups of genes present an obvious challenge to direct interrogation by undirected screens. A further complication is that even in the mouse, few phenotypes are defined with sufficient precision to consistently detect small changes if one or two genes make an incremental contribution to the trisomic phenotype.

Ultimately, it may be less important to tease out “sub-phenotypic” consequences of individual genes than to identify the pathways and processes that are perturbed by trisomy. Correcting unbalanced pathways, regardless of the precise genetic cause, is a logical approach to attenuation of the phenotypic consequences [21].

#### Allelic variation on Hsa21.

Dosage sensitivity may be manifested in another fashion. Allelic variants of Hsa21 genes are present in different ratios in an individual with trisomy than in the diploid state. In the case where a mutant allele results in lower levels of gene product, this mutation will display recessive inheritance when the presence of one wild-type allele is sufficient to carry on normal function. A trisomic condition resulting in two copies of the loss-of-function mutation plus one wild-type copy would probably not alter the phenotypic outcome in this case. However, a recessively inherited phenotype can also occur when a mutant allele produces a gain or change of function, one copy of which does not produce a detrimental effect in the presence of a single wild-type allele, but two copies of which may be sufficient to “overcome” the buffering of a normal allele in a trisomic individual.

Another possible manifestation of trisomy at the molecular level is heterotrisomy, in which alleles from three grandparents are present in every cell [22]. This will occur when trisomy results from an error in meiosis I, the most frequent origin of the extra chromosome in DS [3]. For multimeric proteins assembled from multiple peptides, such as the collagens, the combinatorial possibilities become large. (*COL6A1, COL6A2,* and *COL18A1* are all encoded on distal Hsa21.) Individuals with trisomy will produce combinations of multimers that cannot occur in euploid individuals. Baptista et al. described a region of Hsa21 between *D21S167* and *HMG14* that was frequently heterotrisomic in individuals with DS and CHD [22].

Both “recessive dosage” and heterotrisomy should be amenable to genetic analysis. However, standard statistical methods do not account for the possibility of three alleles in one individual. Sherman, Feingold, and colleagues have established statistical methodologies for genetic association studies to identify genes that affect the DS phenotype when triplicated [23].

#### Coincident small effects.

The preceding examples describe situations in which phenotype is altered due to increased expression of one or a few trisomic genes of major impact. However, small coincident or additive effects of the many genes over-expressed in every trisomic cell may also contribute to trisomic phenotypes. Recent observations confirm that transcript levels are elevated about 1.5-fold for the majority of trisomic genes in a few tissues from humans with trisomy 21 [24,25] and across a broad range of tissues that can be measured in trisomic mouse models [11,12].

In this model, an individual triplicated gene might have no demonstrable impact on phenotype by itself, whereas the collective effect of dozens of genes affecting multiple cellular processes is sufficient to result in a significant impact on phenotype (see [26,27]). Proving this model presents significant experimental challenges, but might be approached by considering quantifiable phenotypes in animal models. Several phenotypes have been measured precisely in trisomic mouse models, allowing comparison between the Ts65Dn mouse, with about 130 Hsa21 orthologs in three copies, and Ts1Cje mice (91 triplicated genes). Behavioral, structural, and functional brain phenotypes, dysmorphology of the skull, and gene expression in the cerebellum all show patterns in Ts65Dn that are similar but attenuated when fewer genes are trisomic in Ts1Cje [6,28–31]. Attenuation of the phenotype when fewer genes are triplicated is consistent with (but not proof of) additive small effects by neighboring trisomic genes.

Note that not only trisomic genes show altered expression in tissues from individuals with trisomy. In some but not all studies, the perturbation of gene expression levels has been demonstrated to extend beyond trisomic genes to those that are disomic, affecting expression levels of a substantial proportion of transcripts in trisomic tissues in mice [32,33]. In one study of trisomic mouse cerebellum, up to one-third of disomic gene transcript levels were subtly altered [32]. Very few of these genes showed a statistically significant difference with euploid when considered individually, but collectively, disomic gene expression robustly distinguished the trisomic and euploid cerebellar transcriptomes. Conflicting results regarding the question of perturbation of disomic gene expression have been reported in human studies [24,25,34]. The controversy is perpetuated by the use of different analytical approaches for array analysis in different studies.

#### Modifier genes.

Most of the features that occur frequently in DS are variable in severity (expressivity) and, except for a few characteristic phenotypes, in occurrence (penetrance). None of the commonly described DS phenotypes are unique to DS or other chromosomal abnormalities but also may occur in euploid individuals [35]. This wide degree of variation suggests that a particular phenotype in a given individual is affected by genetic and environmental variation, and it is reasonable to assume that genetic background (the specific allele set inherited by an individual) affects the severity of outcome.

Preliminary data support the supposition that genetic modifiers contribute to CHD, for which trisomy 21 is the largest risk factor. About half of all individuals with DS have some form of CHD, and most of these involve septal defects. Complete atrioventricular canal occurs in one of five individuals with trisomy 21, compared to 1/10,000 in the euploid population [4]. However, since 80% of those with DS do not have complete atrioventricular canal and 50% have no clinical presentation of heart defects, trisomy 21 is not sufficient to cause CHD by itself. Evidence from patient studies now links the occurrence of CHD in individuals with DS to mutations in disomic genes known to affect septation in mouse models and in non-syndromic atrioventricular canal (C. Maslen, S. Sherman, G. Capone and R. Reeves, unpublished data).

The increased frequency of several important medical conditions in DS, including CHD, childhood onset leukemia, and Hirschsprung disease, suggests that additional genetic factors may be involved. That is, the occurrence of these diseases is greatly increased—but not caused—by trisomy 21. Predisposing modifier genes may combine with the effects of trisomy 21 to reach a threshold effect, resulting in an observable phenotype (see Box 1). Genetic studies in “sensitized” DS populations can be especially effective for identifying genetic variation that contributes to these conditions, regardless of ploidy.

Not all predisposing conditions caused by trisomy have a negative impact. Individuals with DS have reduced frequencies of solid tumors [36,37] and may have a lower incidence of atherosclerosis as well [38,39]. Characterization of these effects could indicate approaches to reducing cancer incidence or cardiovascular disease in all people.

## The Fourth Developmental Dimension: Time

**Figure 2:**
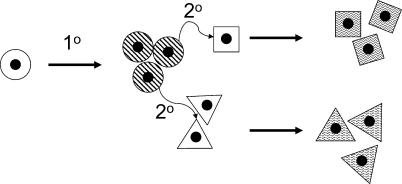
A Primary (1°) Effect of Trisomy Produces an Aberrant Phenotype as the Cells Proliferate Trisomy causes a primary defect in (circular) cells as they proliferate. This primary defect results in a signaling error to neighboring (square and triangular) cells, resulting in their aberrant development as a secondary (2°) consequence of trisomy. Plain background indicates normal cells; striped background indicates an aberrant phenotype.

The demonstration that transcripts of trisomic genes are elevated about 50% in a variety of cells and tissues and at several developmental stages is a reasonable indicator that, for the most part, this level of over-expression will occur in all cells where that gene is expressed throughout development. For those genes whose elevated expression alters a function in fully differentiated cells, the presence of elevated expression in adults may be considered directly in determining the mechanism by which over-expression of that gene contributes to a phenotype of DS. However, over-expression of a given gene will not necessarily affect development and function in every cell type and at every developmental time point when it is expressed at elevated levels. It is likely that over-expression of some genes is detrimental only at a specific time during development, and then only in a specific cell type. Further, a trisomy-induced change in one cell population could affect neighboring cells, resulting in aberrant development as a secondary consequence of trisomy (Figure 2).

For example, in a case where a threshold signal by ligand is required to trigger a step in differentiation, elevated expression of a cell-surface receptor encoded by a trisomic gene could result in the cell experiencing that threshold at a lower concentration of ligand than would be required for a euploid cell. This might cause an early differentiation of cells that would otherwise undergo additional cell divisions before differentiating, resulting in a smaller anlage for subsequent morphogenesis as a primary consequence of trisomy. If this now depauperate cell population normally produces a ligand to signal adjacent cells, the diminished signal produced by fewer cells could have further consequences secondary to those initiated by trisomy. Processing of transcripts or protein can be differentially regulated throughout development as well (e.g., stage-specific switches to alternative splice forms of a message or different phosphorylation states of a variety of proteins). Small alterations of almost any cellular process as a result of aneuploidy could contribute to deviations from normal patterns of development. In particular, dosage effects of regulatory genes could have a wide range of effects [40].

## Ameliorating Consequences of Trisomy 21

Defining the etiology of genetic mechanisms in DS requires knowledge of the trisomic genes, their expression patterns in time and space, and their downstream effects, direct and indirect, on the expression of other genes. This information must be linked to a precise description of phenotypic consequences, not only in fully differentiated cells, but also at all stages where euploid and trisomic developmental processes diverge. Animal models, including critically important segmental trisomies and monosomies in mice, provide a substrate for testing hypotheses about how over-expression of genes individually or in concert can affect development. The precision with which a phenotype and its etiology can be explained in mice points to a difficulty with extrapolation to humans, where phenotypes are defined clinically for practical applications, and not necessarily with the precision required for genetic studies.

Recent advances suggest that the origins of trisomic phenotypes are perhaps even more complicated than assumed for many decades. What then is the most effective way to understand and, more importantly, to ameliorate the effects of trisomy 21 on development and function? No single approach will uncover the myriad sources of divergence from normal development and function initiated by trisomy. One area of research that may be currently under-represented is an approach based in defining etiology—essentially, the interface of genotype and phenotype.

As an example, Ts65Dn mice were shown to have a disproportionately small cerebellum, comparable to a broadly defined phenotype of DS [41]. Closer examination of the trisomic mice demonstrated decreased neuronal density in the Purkinje and granule cell layers, and this new aspect of DS pathology was confirmed in humans. This phenotype was followed through development in mice to identify the earliest stage at which trisomic and euploid cerebellar development diverged. While the number of granule cell precursors was the same at the day of birth, the number of mitotic cells was significantly reduced in trisomic mice. Genetic marker crosses and primary culture assays identified a deficit in the mitogenic response of granule cell precursors to the mitogen, Sonic hedgehog. Treatment with an agonist of the hedgehog pathway corrected the granule cell deficit through (at least) the first third of cerebellar development [42]. This “phenotype-based” approach identified the basis for a method to ameliorate structural deficits in cerebellum and perhaps other brain regions, even though the Mmu16 gene or genes responsible for the mitogenesis response deficit remain to be identified.

## Conclusions

Trisomy 21 is among the most complex genetic conditions compatible with substantial survival beyond birth. This complexity reflects a variety of genetic mechanisms, and the sheer number of genes involved suggests that the primary consequences of trisomic gene over-expression will be amplified throughout development. Ameliorative strategies for DS can be profitably pursued by studying the interface of developmental processes and genetic mechanisms in order to understand the etiology of processes that diverge as a consequence of trisomy.

## Abbreviations

AMKL: acute megakaryoblastic leukemia
CHD: congenital heart disease
DS: Down syndrome
DSCR: Down syndrome critical (or chromosomal) region
Hsa21: human Chromosome 21
Mmu16: mouse Chromosome 16
TMD: transient myeloproliferative disorder

## Notes

Box 1. Trisomy 21 and *GATA1*
The incidence of childhood onset leukemia is elevated in DS and acute megakaryoblastic leukemia (AMKL) occurs almost 500-fold more often than in the euploid population [43]. This is associated with an unusual feature of DS, transient myeloproliferative disorder (TMD). About 10% of newborns with DS exhibit TMD, an expansion of immature megakaryoblasts [44] that usually undergoes spontaneous remission shortly after birth without clinical consequences. However, 20%–30% of those who have TMD will develop AMKL later in life. Somatic mutations in the *GATA1* transcription factor gene, encoded on the X chromosome, occur in most AMKL and almost every case of TMD in persons with DS, but not in other leukemias that occur in DS; further, *GATA1* mutations have never been seen when AMKL occurs in euploid individuals except when the expanded blasts are trisomic for Hsa21 [45]. Thus the relationship between trisomy 21 and *GATA1* is complex. It appears that trisomy 21 makes megakaryoblasts highly sensitive to *GATA1* mutations. The same mutations presumably occur in euploid individuals, but have no deleterious consequence unless Hsa21 becomes triplicated in those cells. It would be interesting to know if the frequency of *GATA1* mutations is the same in megakaryoblasts trisomic for Hsa21 as it is in euploid blasts.
